# The complete chloroplast genome of *Forsythia mira*, an endemic medicinal shrub in China

**DOI:** 10.1080/23802359.2019.1693924

**Published:** 2019-12-09

**Authors:** Su Gao, Ji-Qing Bai, Mi-Li Liu, Peng-Fei Wang, Na Li, Lei Yang, Xiao-Ping Wang, Zhong-Hu Li

**Affiliations:** a College of Pharmacy, Shaanxi University of Chinese Medicine, Xianyang, China;; b Shaanxi Quality Monitoring and Technology Service Center for Chinese Materia Medica Raw Materials, Xianyang, China;; c Key Laboratory of Resource Biology and Biotechnology in Western China, Ministry of Education, College of Life Sciences, Northwest University, Xi’an, China

**Keywords:** *Forsythia mira*, chloroplast genome, phylogenetic tree

## Abstract

*Forsythia mira* M. C. Chang (Oleaceae) is an endemic medicinal shrub in China. In this study, we first characterized its whole plastid genome sequence using the Illumina sequencing platform. The plastid genome was 156,485 bp in length, comprising of a large single copy (LSC) region of 87,223 bp, a small single copy (SSC) region of 17,830 bp, and two inverted repeat regions (IRs) of 51,432 each. The genome of *F*. *mira* contained 133 genes, including 86 protein-coding genes, 37 transfer RNAs (tRNA), and 8 ribosomal RNAs (rRNA). The phylogenetic analysis showed that *F*. *mira* was placed as a sister to the congeneric *F*. *suspensa*.

*Forsythia mira* M. C. Chang (Oleaceae) is an important medicinal shrub species, only distributed in Shanyang country, Shaanxi Province, China. It is commonly known as Lian-Qiao in the Traditional Chinese Medicine, and the fruit of species is used locally as a very important medicine, which have some pharmacological effects, such as anti-inflammatory, antioxidant, antibacterial, antivirus, anticancer, and anti-allergy effects (Wang et al. 2018). However, there are few reports about the evolutionary aspects of *F*. *mira*. Herein, we performed high-throughout sequencing of the chloroplast (cp) genome of *F. mira*.

The fresh leaves samples of *F. mira* were collected from Shanyang county, Shaanxi province, China (N33.559541, E109.740769). The specimen (QY-19082104) is stored in the Shaanxi University of Chinese Medicine. Total genomic DNA of *F. Mira* from a single individual was isolated by a modified CTAB method (Doyle and Doyle 1987). Then, the DNAs were subjected to Illumina sample preparation, and pair-read sequencing was indexed by the Illumina HiSeq 2500 platform. The high-quality reads were obtained, and the clean reads were assembled by the MIRA 4.0.2 program (Chevreux et al. 2004) and MITObim version 1.7 (Hahn et al. 2013). Annotation of the cp genome with GENEIOUS R8 (Fan et al. 2016) was manually adjusted by comparison with homologous genes of *F*. *suspensa* cp genome (NC_036367.1). Finally, the annotated cp genome of *F*. *mira* was submitted to GenBank (accession number: MN560167) and the circular genome maps were drawn using OGDRAW (Liu et al. 2018; Wang et al. 2019).

The complete cp genome of *F*. *mira* was a typical quadripartite circular molecule with a length of 156,485 bp, comprising a large single copy (LSC) region of 87,223 bp and a small single copy (SSC) region of 17,830 bp, separated by two inverted repeat regions (IRs) of 51,432 bp. The circular genome contained 133 genes, including 86 protein-coding genes, 37 tRNA, and 8 rRNA genes. Most gene species occurred in a single copy, while 20 gene species occurred in double copies. The overall GC content of *F*. *mira* chloroplast genome was 37.8%, while the corresponding values of LSC, SSC, and IR regions were 35.8%, 31.8% and 43.2%, respectively.

A total of 23 species cp genomes downloaded from NCBI were used to construct the phylogenetic tree. The plastid sequences were aligned using the software MAFFT (Katoh and Standley 2013) with the default parameters. The phylogenetic analysis was conducted using the program MEGA X (Li et al. 2019) with 1000 bootstrap replicates. The results indicated that *F*. *mira* was placed as a sister to the congeneric *F*. *suspensa* (Figure 1).

**Figure 1.**
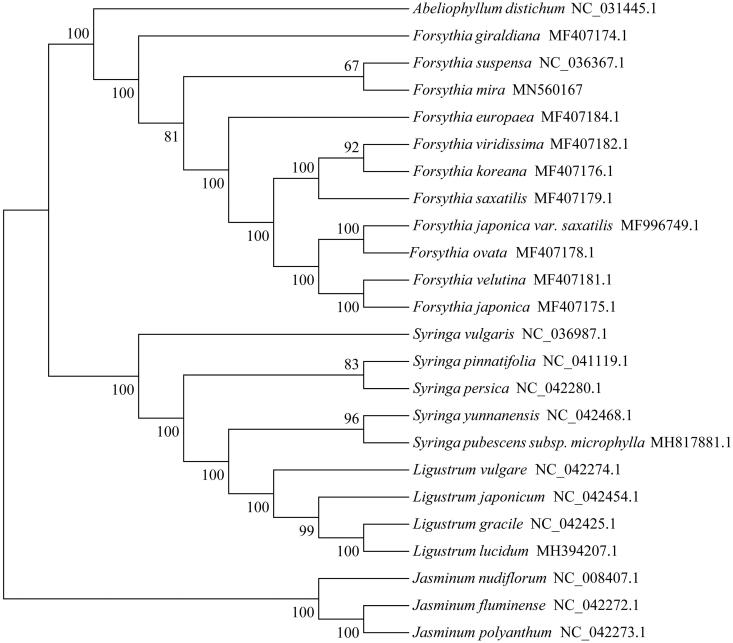
Phylogenetic tree based on 24 complete chloroplast genome sequences.
